# The sequential phosphorylation of PHF10 subunit of the PBAF chromatin-remodeling complex determines different properties of the PHF10 isoforms

**DOI:** 10.1242/bio.043943

**Published:** 2020-01-15

**Authors:** Andrey A. Sheynov, Victor V. Tatarskiy, Eugene V. Tatarskiy, Elena N. Nabirochkina, Sofia G. Georgieva, Nataliya V. Soshnikova

**Affiliations:** Department of Eukaryotic Transcription Factors, Institute of Gene Biology, Russian Academy of Sciences, Vavilov Street 34/5, Moscow 119991, Russia

**Keywords:** PBAF chromatin remodeling complex, PHF10 isoforms, Multiple phosphorylation, Cluster phosphorylation

## Abstract

The mammalian PBAF subfamily of SWI/SNF chromatin remodeling complexes plays a wide role in the regulation of gene expression. PHF10 is a subunit of the signature module of PBAF, responsible for its interaction with chromatin. PHF10 is represented by four different isoforms, which are alternatively incorporated in the complex. Two of PHF10 isoforms lacking C-terminal PHD domains contain a cluster of phosphorylated serine residues, designated as X-cluster. In the present study, we explore the phosphorylation of the X-cluster in detail. We identified additional phosphorylated serine residues and designated them as either frequently or rarely phosphorylated. The X-cluster consists of two independently phosphorylated subclusters. Phosphorylation of the second subcluster depends on phosphorylation of a primary serine 327. These two subclusters surround a sequence, which is predicted to be a nuclear localization sequence (NLS3). The NLS3 does not affect localization of PHF10 isoforms. However, it is essential for X-cluster phosphorylation and increased stability of isoforms that lack PHD. Conversely, the presence of NLS3 signal in isoforms that contain C-terminal PHD domains reduces their stability. Thus, phosphorylation of PHF10 isoforms regulates their cell level, determining the rate of incorporation in PBAF. This may alter the pattern of PBAF regulated genes.

## Abstract (precis)

**Summary:** The sequential phosphorylation of the linker domain of PHF10 subunit of PBAF chromatin remodeling complex is triggered by two primarily phosphorylated serines and determines the different properties of PHF10 isoforms.

## INTRODUCTION

Phosphorylation is the most widespread and the most studied post-translational modification in eukaryotes. Phosphorylation is involved in regulation of various cellular processes like transcription and catalytic activity; it can alter protein structure, conformation, stability, localization and protein interactions (Nishi et al., 2014; Ubersax and Ferrell, 2007). Phosphorylation allows for an additional level of complexity and regulation. Both singular proteins and proteins inside complexes can be phosphorylated, which in turn can affect the function of the whole complex.

Sites of phosphorylation in many proteins tend to form clusters with many closely located amino acids phosphorylated by the same kinase. Importantly, the phosphorylation events in clusters tend to be coordinated with phosphorylation of each amino acid, determined by the phosphorylation of the previous one (Freschi et al., 2014; Li et al., 2009, 2017; Schweiger and Linial, 2010).

Phosphorylation is likely to be important for the regulation of the mammalian PBAF subfamily of SWI/SNF chromatin-remodeling complex, which plays an important role in the regulation of transcription (Simone, 2006; Brechalov et al., 2016). The PBAF complex consists of core subunits including ATPase (Brg1 or Brm) and signature subunits (BAF180, BRD7 and BAF200, and PHF10/BAF45A), which are alternatively incorporated in complex and are essential for complex interaction with chromatin (Moshkin et al., 2007). Several of the core PBAF proteins, including BAF155, BAF170, BAF47 and BAF60c are phosphorylated. BAF155, BAF170 and BAF47 interact with and are phosphorylated by the Akt kinase, and these modifications regulate genes of the Akt/PI3K signaling pathway and cell replication (Foster et al., 2009).

Among the signature subunits of PBAF, only PHF10 was shown to be extensively phosphorylated (Tatarskiy et al., 2017). PHF10 is the highly important subunit of PBAF (Brechalov et al., 2014; Lessard et al., 2007) that is essential for PBAF interaction with target genes. PHF10 is represented in mammalian cells by four isoforms, which differ in their N- and C-end domains (Brechalov et al., 2014), are alternatively incorporated in PBAF and have different gene targets. The incorporation of different PHF10 isoforms into PBAF alters the functions of the whole complex and thus is important for changing the activity of PBAF (Brechalov et al., 2016).

In previous work, we have shown that each PHF10 isoform has its own unique phosphorylation pattern that is dependent on its domain structure (Tatarskiy et al., 2017). The important role of phosphorylation is to maintain the different level of stability of PHF10 isoforms in the cell that, in turn, affects their presence in the complex. We have demonstrated that PHF10 isoforms that lack C-terminal PHD domains (PHF10-S) are much more stable in the cell than isoforms that contain PHD (Brechalov et al., 2016; Tatarskiy et al., 2017). Serines 297, 301, 327 and 331 of the linker domain of the PHF10-S isoforms (designated as X-cluster) overlap with β-TrCP degrons and are phosphorylated by casein kinase 1 (CK1), which prevents the interaction with b-TrCP-ubiquitin-ligase and subsequent degradation (Tatarskiy et al., 2017).

In the present study, we expanded our understanding of the structure and function of X-cluster and its role in maintaining the stability of the PHF10-S isoforms. We demonstrate that the phosphorylation events in X-cluster are highly coordinated. The X-cluster consists of two independently phosphorylated sub-clusters that surround a potential nuclear localization signal (NLS3). The first sub-cluster contains two primary phosphorylated serines 297 and 301. The second sub-cluster includes the primary phosphorylated serine 327, its phosphorylation triggers phosphorylation of serines 331, as well as serines 323 and 335, which were also found to be phosphorylated. The potential NLS signal does not target the PHF10-S isoforms to the nucleus but is required for phosphorylation of surrounding amino acids. These findings show a complex mechanism for regulation of PHF10 stability, through highly modified novel domains.

## RESULTS

### The X-cluster of PHF10 contains six serine amino acids, which are phosphorylated with different levels of probability

**Fig. 1.**
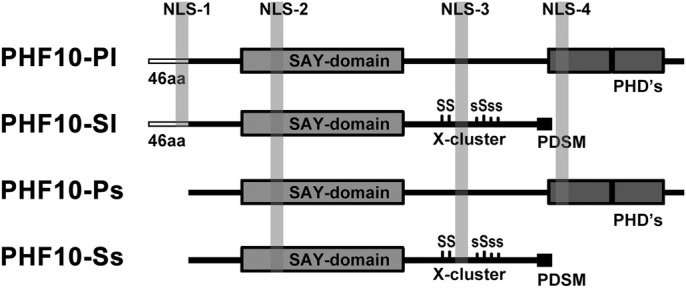
**Domain organization and localization of the X-cluster and nuclear localization sites (NLS) within PHF10 isoforms.** N-terminal domain, SAY-domain, linker domain and double PHD or PDSM domains are indicated. NLS-2 and NLS-3 are common for all PHF10 isoforms, NLS-4 is specific for PHF10-P isoforms. Serines of X-cluster are phosphorylated in PHF10-S isoforms.

The domain structure of PHF10 is shown in (Fig. 1). The isoforms differ by the domain structure of their N- and C- ends, which determines the possible phosphorylation modifications. The absence of C-terminal PHD domains in two isoforms (PHF10-Sl and PHF10-Ss) allows phosphorylation of their linker domain by casein kinase 1 (CK1) (X-cluster phosphorylation), which is not possible in isoforms that contain PHD domains on the C-end (Tatarskiy et al., 2017). One of the three predicted nuclear localization signals (NLS3) is localized in the X-cluster (Fig. 1).

**Fig. 2.**
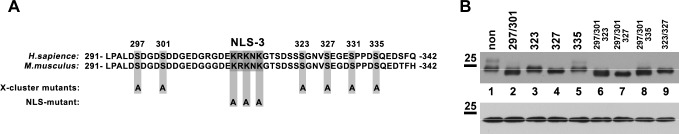
**Serines 297/301 and 327 are phosphorylated independently of each other and organized in two independent subclusters.** (A) Partial sequence of the linker domain. Serines of X-cluster are highlighted and NLS-3 is marked by a grey box. Replacement of serines by alanines in the X-cluster mutants and lysines with alanines in the NLS-mutants is indicated by light grey boxes. (B) The FLAG-tagged PHF10 linker domain and its mutated X-cluster forms were overexpressed in HEK293 and then immunoblotted with anti-FLAG antibodies (upper panel). The lower panel represents the same forms of the linker domain overexpressed in HEK293 and treated with λ-PP before loading to the PAGE. The horizontal white line is overlaid to make comparison of band mobility easier.

Previously we investigated phosphorylation of the X-cluster and showed (Tatarskiy et al., 2017) that CK1 phosphorylates four serine amino acid residues 297, 301, 327 and 331 (Fig. 2A). Moreover, serines 297, 301 and 327 are phosphorylated more often than serine 331 (Tatarskiy et al., 2017). To supplement this data we analyzed phospho-modifications of this cluster published in high-throughput mass-spectrometry screenings available through the PhosphoSite database (Hornbeck et al., 2015). This database allows comparing the number of high-throughput screenings (HTS) of phospho-modifications in which a given residue in the protein has been modified. Our analysis of PhosphoSite has shown that the X-cluster can also include serines 323 and 335 (Fig. 2A). These serine residues were found in only two screenings, compared to residues 297, 301 and 327, which were found in more than 25 screenings. Phosphorylation of serine 319 and 339 was not detected; however, based on the amino acid motif of the X-cluster they may be also the targets for phosphorylation.

The sequence of the PHF10 linker domain is conservative and in the screenings of mouse and rat sequences the corresponding amino acid residues are also phosphorylated, which indicates their importance (Fig. 2A). We hypothesized that serine residues 297, 301 and 327, which are often detected as phosphorylated, can influence the phosphorylation of nearby serines 32, 331 and 335 (Fig. 2A).

The phosphorylation of serines was first examined in HEK293 cells. We expressed recombinant non-mutated and mutated forms of the FLAG-tagged linker domain in HEK293 cells. In mutated forms, the serines that were the potential phosphorylation targets were replaced by alanine (Fig. 2A). The wild-type and mutated forms of the linker domain had different mobility in PAAG gel (Fig. 2B, upper panel). Treatment with λ-PP completely abolished difference in the mobility of the wild-type and mutated forms of the linker domain (Fig. 2B, lower panel), indicating that different mobility reflected different levels of their phosphorylation.

The wild-type linker domain migrates as two bands in PAAG gel (Fig. 2B, lane 1). The mutations of serine residues 297, 301 and 327 (Fig. 2B, lanes 6 and 7) strongly increased mobility of the linker domain, which now migrated as a single band, confirming that these serine residues were phosphorylated as was shown previously (Tatarskiy et al., 2017). The effect of mutations in residues 323 and 335 on the mobility in PAAG gel was much lower (Fig. 2B, lanes 3 and 5). Still, a small change in the phosphorylation of proteins with mutated serines 323 and 335 was observed, since the upper band corresponding to the completely phosphorylated form of the linker domain was absent in the mutant for serine 323 and became weaker in the mutant for serine 335. These data indicate that serine 323 and 335 were also phosphorylated *in vivo*. However, the level of their phosphorylation was much lower than that of serines 297, 301 and 327.

**Fig. 3.**
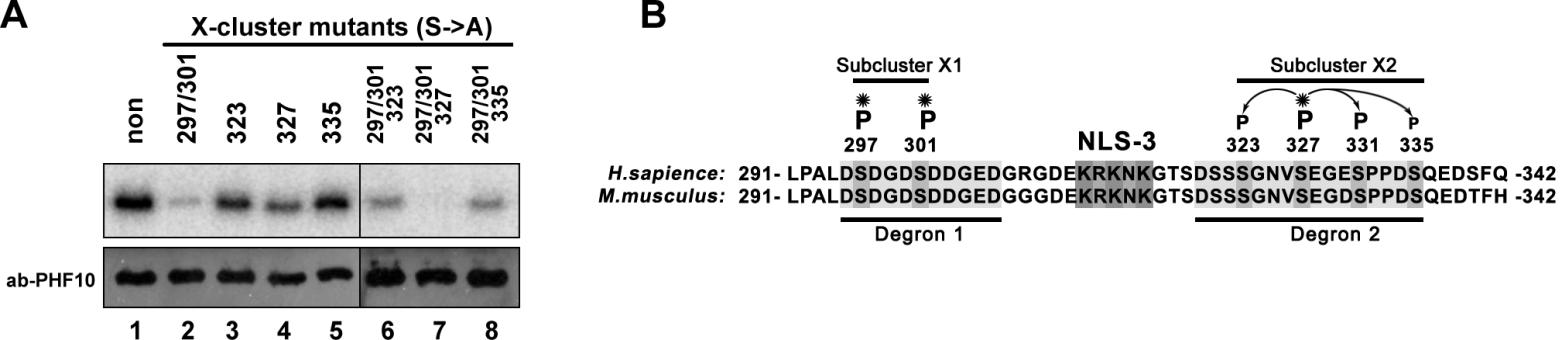
**Phosphorylation of serine residue 327 primes serines 323, 331 and 335 for phosphorylation.** (A) The 6His-tag PHF10 linker domain (amino acids 291–342), and its mutated variants were expressed within *E. coli* system, purified and incubated with HEK293 extract supplied with Gamma-[31] P-ATP. Different mutated forms of the linker domain have a different level of the signal that depends on the strength of the phosphorylation site. The purified and immunostained 6His-linker domain of PHF10 was used as the loading control. (B) A partial sequence of the linker domain. Serines of the X-subclusters-1 and -2 are highlighted and NLS-3 is marked by a grey box and marked by horizontal black lines at the top. B-Trcp Degrons-1 and -2 are also highlighted and marked by horizontal black lines below the sequences. Priming serines 297, 301 and 327 are marked by asterisks and arrows from serine 327 point to adjacent phosphorylated serines 323, 331 and 335.

Next, to examine the phosphorylation of studied serines we performed a kinase assay. The linker domain of PHF10 as well as its mutated variants were expressed in bacteria, purified using His-tag columns and incubated with HEK293 lysates in the presence of [P]-ATP (Fig. 3A). We detected a significant decrease in the signal when 297 and 301 serine residues were mutated. The signal was also partially decreased when the 327 serine was mutated, while mutations in 323 and 335 serine residues showed only a small decrease, which correlates to its proposed low phosphorylation level (Fig. 3A; compare lanes 3 and 5 with line 1). Thus, in this *in vitro* system, we have confirmed that in addition to the frequently phosphorylated serines 297, 301 and 327, the X-cluster contains serines 335 and 323 that are phosphorylated at lower levels.

### Serines 297/301 and 327 are phosphorylated independently of each other

To increase signal transduction through phosphorylation, phosphorylated residues could be organized in clusters (Schweiger and Linial, 2010). Phosphorylation of amino acids within the cluster occurs as a sequence, initiated by the phosphorylation of one serine, which is required to start the cascade (Li et al., 2009, 2017; Schweiger and Linial, 2010). In the present case, phosphorylated serines are organized into two sub-clusters that surround a sequence, which contains a signal of nuclear localization.

To determine whether phosphorylation of serines in the X-cluster depend on each other we first examined the frequently phosphorylated serines 297, 301 and 327. We produced recombinant forms of the linker domain, which contained mutations of serines in the first sub-cluster (297/301), or in the second (327). The signal in kinase assay decreased only partially when serines of only one sub-cluster were mutated, while the simultaneous mutation of serines 297/301 and 327 completely abolished phosphorylation (Fig. 3A; compare lines 1 with 2, and 4 with line 7). This means that serine residues 297/301 and 327 are phosphorylated independently from each other.

Analysis of electrophoresis mobility of non-mutated and mutated FLAG-tagged linker domain showed that mobility of the linker domain with mutations of all 297, 301 and 327 serines was lower than for each of mutants separately. This also confirms that serines 297/301 and 327 are phosphorylated independently (Fig. 2B; compare line 7 with lines 2 and 4). In summary, it confirms that the X-cluster of PHF10 contains two independently phosphorylated sub-clusters.

### Phosphorylation of serine residue 327 primes serines 323, 331 and 335 for phosphorylation

The frequently phosphorylated serine 327 in the second sub-cluster is surrounded by rarely phosphorylated serines 323, 331 and 335. By comparing their phosphorylation in kinase assay and electrophoretic mobility in a gel, we determined if their phosphorylation was dependent on phosphorylation of serine 327.

As we expected, the kinase assay showed that mutations of the 323, 331 and 335 serines had no effect on phosphorylation of serine 327. They also had no effect on 297/301 serines of the other sub-clusters (Fig. 3A; lines 3 and 5). In turn, phosphorylation of serines 323, 331 and 335 in the second sub-cluster did not depend on phosphorylation of the serines 297/301 in the first sub-cluster (Fig. 3A; lines 2, 6 and 8). Only an additional mutation of serine 327 leads to the full absence of phosphorylation (Fig. 3A; lines 7 and 8; in Fig. 2B, lines 2, 6 and 8 are similar). Thus, phosphorylation of serines 323, 331 and 335 probably depends on phosphorylation of serine 327.

To confirm this result we expressed the linker domain in HEK293 cells (Fig. 2B) and determined its mobility in SDS-PAGE. The mutations of serines 297/301 of the first sub-cluster increased its electrophoretic mobility (Fig. 2B; compare line 1 and 2), indicating a decrease in phosphorylation. Mutation of frequently phosphorylated serines resulted in a protein with the greatest electrophoretic mobility (Fig. 2B; line 7) represented by only one form, indicating total loss of phosphorylation.

Mutation of serines 297/301 of the first sub-cluster resulted in only partial differences compared to the non-mutated fragment (Fig. 2B; compare lines 1 and 2), as faint bands could be detected above the main (non-phosphorylated) band. These faint bands corresponded to phosphorylated serines 323, 331 and 335 and disappeared when these amino acids were mutated (Fig. 2B; lines 3, 5, 8 and 9). It is noteworthy that they disappeared if serine 327 from the second sub-cluster was mutated alone or together with the mutations of serines 293 and 301 (Fig. 2B; lane 7). Therefore, the serine 327 of the second sub-cluster was the priming amino acid required for phosphorylation of serines 323, 331 and 335.

### NLS3 does not determine the localization of PHF10 but is important for its degradation

The two phosphorylation sub-clusters surround the motif, which is predicted to be a sequence for nuclear localization (NLS3). The nuclear localization sequences usually contain several positively charged amino acids (most frequently arginine and lysine), which are recognized by importin proteins. Phosphorylation of the X-cluster creates a strong negative charge on both sides of the NLS3 and therefore can mask NLS3 and prevent its recognition by importins (Fig. 2A).

First, we tested whether NLS3 sequence was functional and influenced the localization of isoforms. To this aim, we mutated the NLS3 in one of the PHDs containing PHF10 isoforms in which Х-cluster is not phosphorylated (PHF10-Ps). In mutated FLAG-tagged PHF10-Ps lysines in the NLS3 sequence were replaced by neutral alanines (Fig. 2A).

**Fig. 4.**
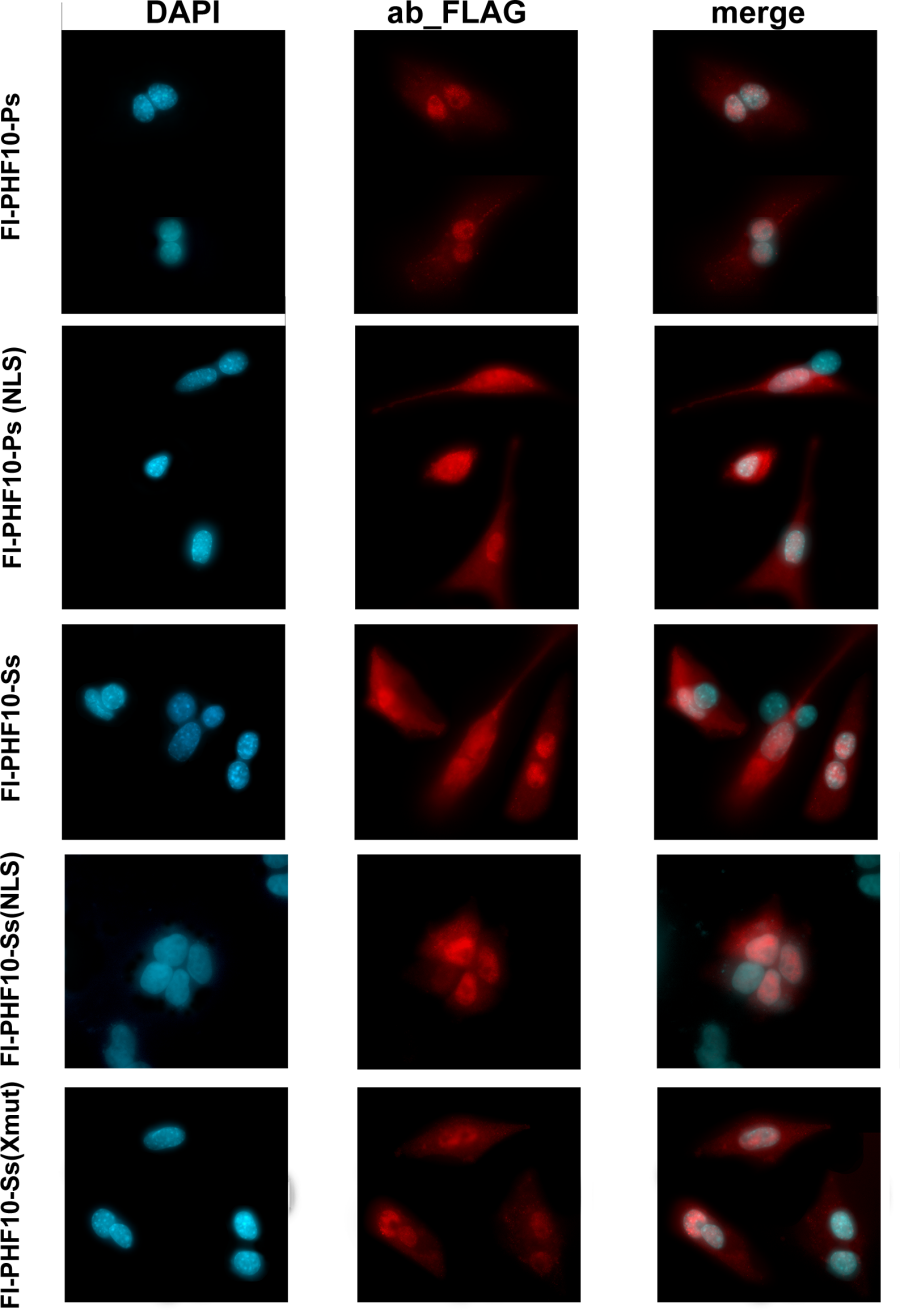
**Immunostaining of FLAG-PHF10-Ps/-Ss and X-cluster/–NLS mutants.** Transiently expressed for 24 h in HEK293 cells, isoforms were stained with anti-FLAG antibodies. Both PHF10-Ps and PHF10-Ss isoforms are localized in the nucleus and cytoplasm. NLS mutation did not affect the localization of PHF10-Ps and PHF10-Ss isoforms. PHF10-Ps, PHF10-Ss NLS- and -X-cluster mutants are expressed more weakly than PHF10-Ps(NLS) and PHF10-Ss wild-type isoforms.

**Fig. 5.**
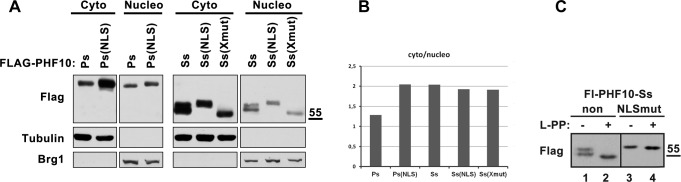
**Subcellular localization of FLAG-PHF10-Ps/-Ss and X-cluster/–NLS mutants.** (A) HEK293 cells were transfected with FLAG-PHF10-Ps/Ss isoforms and their mutated variants. After 24 h cells were fractionated to cytoplasmic and nuclear fractions. Protein extracts were analyzed by western blotting. BRG1 and β-tubulin were used as fractionation and loading controls. (B) The intensity of bands on the western blot was quantified using ImageJ software by densitometry, as described in the Materials and Methods section. The ratio of cytoplasm and nucleus fractions of PHF10-Ps isoform is approximately equal, a mutation in NLS-3 lead to accumulation of PHF10-Ps(NLS) in the cytoplasm. FLAG-PHF10-Ss and its mutated forms have the same ratio between cytoplasm and nucleus, but the reduction of phosphorylation in the X-cluster (in X-mut and NLS mut) leads to decreased stability of PHF10-Ss isoform. (C) Western blot of FLAG-PHF10-Ss non mutated and FLAG-PHF10-Ss(NLS) isoforms treated by λ-PP for the elimination of phosphorylation. The FLAG-PHF10-Ss and its mutated form NLSmut were visualized using anti-FLAG antibodies on SDS-PAGE. The difference in the mobility of NLSmut compared to FLAG-PHF10-Ss was caused by a change of positively charged lysines into neutral alanines in the mutant. We detected a strong band shift in case of PHF10-Ss(non) and no band shift in case of PHF10-Ss(NLS).

The endogenous PHF10-Ps, as well as its FLAG-tagged form, was present in both nuclei and cytoplasm. After overexpression of mutated Fl-PHF10-Ps-NLS, we did not detect any changes in the nuclear protein levels, indicating that the NLS3 did not affect translocation into the nucleus. Surprisingly, the amount of the mutated protein in the cytoplasm increased significantly (Fig. 4). Similar results were obtained after fractionating cell extracts into nuclear and cytoplasmic fractions (Fig. 5A,B). Thus, mutation of NLS3 sequence leads to accumulation of mutated PHF10-Ps isoform in cytoplasm pointing to its increased stability. This indicates that rather than affecting translocation, this sequence is required for degradation. In summary, the NLS3 sequence is a functional part of the protein and involved in its degradation in the cytoplasm.

### Phosphorylation of the X-cluster does not affect the localization of PHF10-Ss but prevents its degradation in the cytoplasm

We further suggested that X-cluster phosphorylation in isoforms lacking PHDs can affect the function of NLS3. Similar to PHF10-Ps, the endogenous PHF10-Ss isoform lacking PHDs was detected in both nuclei and cytoplasm, but the content of PHF10-Ss in the cytoplasm was much higher than that of PHF10-Ps (Figs 4 and 5A,C).

We expressed the PHF10-Ss isoform with mutated serines 297/301 and 327 of X-cluster eliminating its phosphorylation [Ss(Xmut)] and performed immunostaining experiments. As expected, the phosphorylation was completely lost in mutated PHF10-Ss and it migrated as a single band on SDS-PAGE. Both fluorescent microscopy and western blot experiments showed a decrease of PHF10-Ss levels in the cytoplasm and in the nucleus (Fig. 5B), confirming that the absence of X-cluster phosphorylation significantly decreases the stability of PHF10-Ss isoform. However, there were no changes in the distribution of PHF10 between the cytoplasm and the nucleus (Fig. 5B). These results indicate that NLS3 does not target PHF10-Ss to the nucleus, similar to what was shown for PHF10-Ps isoform.

### Mutation of NLS3 eliminates phosphorylation of PHF10-Ss isoform

In order to reveal the function of NLS3 in PHF10-Ss isoform, we mutated the lysine residues in NLS3 sequence, similarly to PHF10-Ps-NLS (Fig. 2A). The distribution of the mutated protein in the cell was studied both by fluorescent microscopy (Fig. 4) and nuclear and cytoplasm fractionation. Both methods showed that the mutation of NSL3 residues did not influence the distribution of PHF10 between cellular compartments. However, it led to a substantial decrease of PHF10 in the nucleus and cytoplasm (Figs 4 and 5A,B). The mutation of NLS3 also significantly decreases the phosphorylation of PHF10-Ss isoform (Fig. 5C).

To better demonstrate that NLS3 is required for the phosphorylation of the surrounding serines of X-cluster, the protein extract from cells transformed with FLAG-PHF10-Ss or its mutant form were treated with Lambda-phosphatase. The effect of Lambda-phosphatase treatment on isoform mobility was analyzed on SDS-PAGE. Incubation of FLAG-PHF10-Ss with Lambda-phosphatase led to a substantial change in its mobility (Fig. 5C; compare lane 1 and lane 2) indicating the high level of FLAG-PHF10-Ss phosphorylation. On the contrary, the NLSmut version did not change its mobility after Lambda-phosphatase treatment (Fig. 5C; lane 3 and 4), which indicates the absence of phosphorylation.

Therefore, NLS3 sequence in PHF10-Ss isoform does not affect its cellular localization but is important for phosphorylation of X-cluster serine residues that are essential to prevent Ss-isoform degradation.

## DISCUSSION

In the present article, we demonstrate that the region in the linker domain of PHF10-S isoforms (X-cluster) consists of two independently phosphorylated clusters of serine residues. We identified the primarily phosphorylated serines and demonstrated that they are essential to trigger the phosphorylation of other serines in the cluster. The two clusters are separated by the predicted NLS (NLS3) sequence. However, our data show that the NLS3 does not target the PHF10 isoforms into the nucleus but is involved in maintaining their phosphorylation and stability. The NLS3 increases the rate of degradation of PHDs containing PHF10 isoforms. In PHF10 isoforms lacking C-terminal PHDs, the NLS3 is essential for phosphorylation and thus is essential for their high stability in the cell. Overall, these data demonstrate the complicated mechanism of maintaining the turnover rate of different PHF isoforms in the cell.

The PHF10 isoforms that lack PHD domains are phosphorylated on six serine residues in the linker domain. The serines in the linker domain of PHF10 (X-cluster) are phosphorylated in PHF10-S isoforms. In turn, the X-cluster consists of two sub-clusters that are independently phosphorylated. The first sub-cluster contains serines 297 and 301, and the second – serines 323, 327, 331 and 335. The two serines of the first sub-cluster and serine 327 of the second sub-cluster are actively phosphorylated, independently of each other. Phosphorylation of serines 323, 331 and 335 depends on phosphorylation of serine 327 and does not happen if this residue is mutated into alanine. Thus, phosphorylation of serine 327 serves as a primer, required for phosphorylation of other serines in the sub-cluster.

In sequentially phosphorylated sites, the phosphorylation of residues happens in a strict order, when phosphorylation of one residue is required for phosphorylation of the next phosphorylated amino acid in the sequence (Salazar and Höfer, 2009). The number of screenings that indicate phosphorylation of serines in the second sub-cluster (327, 27; 331, 3; 323, 2; 335, 2) indicates that the phosphorylation happens sequentially from the 327 serine to the 331, 323 and 335 serines.

In recent publications, triggered sequential phosphorylation is considered an important mechanism of regulation. Such phosphorylation is highly coordinated and could modify the speed and sensitivity of the cellular response to external stimuli (Gunawardena, 2005; Mao et al., 2008). Phosphorylation of multiple sites could also serve as an integrating point for activating and inhibiting signals, which in turn determine proteins’ fate and events in the downstream signaling (Nishi et al., 2014). One example of such a mechanism is DNA-dependent RNA polymerase II. The multiple phosphorylation of the amino acids in the CTD domain of the RNA polymerase II determines the staging of the transcription cycle in which it participates.

Previously we have shown that phosphorylation of 297 and 301 serines, as well as of serines 327 and 331 in the second sub-cluster is performed by the casein kinase 1 (CK1). In line with this data, it was demonstrated that serines and threonines, organized in clusters and phosphorylated by the same kinases, are usually located in unstructured domains (Schweiger and Linial, 2010), and evolutionary clustered sites are 1.4 times more frequently phosphorylated by the same kinase (Freschi et al., 2014). The distance between the sites is critical for their sequential phosphorylation (Kõivomägi et al., 2013).

Previously we have shown that phosphorylation of the X-cluster leads to increased stability of PHF10-Sl and PHF10-Ss isoforms, compared to PHF10-Pl and PHF10-Ps, in which the X-cluster is not phosphorylated. One of the functions of cluster phosphorylation was shown to be a regulation of protein stability and degradation (Landry et al., 2014; Varedi K et al., 2010). In line with these data, X-cluster phosphorylation is also tightly involved in the regulation of PHF10 isoforms turnover rate in a cell. The PHF10-Ps, degrades faster due to the lack of phosphorylation in the X-cluster, while the phosphorylation of the same residues in the PHF10-Ss isoform blocks its interaction with the beta-Trcp and stabilizes it (Tatarskiy et al., 2017). Here we show that NLS3 also stimulates the degradation of PHF10-Ps isoforms. Mutation of the potential nuclear localization site in the PHF10-Ps leads to the accumulation of the corresponding isoform.

In our experiments, the same mutation of the NLS3 motif of PHF10-Ss had the opposite effect, leading to a decreased stability of the protein. However, we have found that this mutation also completely abolished the X-cluster phosphorylation that, as was shown before, protects PHF10-Ss from degradation. It is difficult to clearly understand the way NLS3 motif influences phosphorylation as it is not indicated in databases whether it has any additional functions. One may suggest that altering the charge of mutant NLS3 may influence the secondary structure of PHF10-Ss protein.

Short isoforms PHF10-Ps/-Ss are localized both in the nucleus and in the cytoplasm, but the PHF10-Ss is mostly localized in the cytoplasm. From our data, it became apparent that the difference in the distribution between compartments is determined by the different speed of degradation.

The amount of PHF10-Ps and PHF10-Ss in the nucleus was similar. As we have shown previously (Brechalov et al., 2014) recombinant PHF10-Ps and PHF10-Ss in the nucleus are incorporated into the PBAF complex. Therefore, it is possible that PHF10-Ps isoforms are mainly incorporated into the PBAF complex and are important for its functioning, while PHF10-Ss, due to its localization and increased stability, has functions in the cytoplasm.

Proteins with closely located phosphorylation sites combined into clusters have a limited number of functions. For cytoplasmic proteins, such site organization is typical for cytoskeleton proteins and proteins that regulate translation (Schweiger and Linial, 2010). It is possible that the functions of PHF10-Ss in the cytoplasm could be related to one of these classes. For nuclear proteins, a strong negative charge of such clusters can change electrostatic interactions of such proteins with DNA (Gunawardena, 2005).

In the nucleus, the PHF10-Ss and PHF10-Sl isoforms are also phosphorylated on X-cluster serines. Thus, which isoforms, PHF10-P or PHF10-S, that are incorporated into the complex can determine the interactions of the whole complex with DNA and histones, and the complex functioning during chromatin remodeling.

## MATERIALS AND METHODS

### Cells and extracts

HEK293 cells were grown in DMEM medium supplemented with 10% FBS (HyClone), 2 mM L-glutamine at 37°C, 5% CO_2_. HEK293 cells were lysed in Lysis Buffer: 10 mM HEPES (pH 7.9) containing 5 mM MgCl, 0.5% Nonidet P-40, 0.45 M NaCl, 1 mM DTT, a protease inhibitor cocktail (PIC) (Roche) and a 1% Phosphatase inhibitor cocktail 3 (PhIC) (Sigma-Aldrich). The lysate was centrifuged at 10,000 rpm, 4°C, for 10 min, and the supernatant was diluted fourfold with the same buffer but without NaCl. The extract was treated with DNAse I (Thermo Fisher Scientific; 0.6 units/ml) and RNase (Thermo Fisher Scientific; 10 units/ml).

### Cellular fractionation

Cells were lysed in FLB buffer (40 mM Tris-HCl, pH=7.8; 100 mM NaCl; 2.5 mM MgCl_2_, 1 mM DTT; PIC; PhIC) on the ice, ground in Loose Dounce, centrifuged for 1 min and the supernatant was employed as the cytoplasmic fraction. The pellet was resuspended in Lysis Buffer, ground in Tight Dounce, incubated for 10 min on ice, centrifuged as mentioned above and diluted the same way. The probes were equalized using Qubit Protein Assay Kit (Thermo Fisher Scientific), mixed with 4X LB (200 mM Tris-HCl, pH=6.8; 4% SDS; 40% Glycerol; ∼0.01% Bromophenol Blue; 100 mM DTT), and boiled for 10°C.

### Kinase assay

*In vitro* kinase assay was performed as described previously (Tatarskiy et al., 2017). For *in vitro* kinase assay linker domain (238-361 aa), X-cluster serine mutants and NLS-3 mutant forms were cloned in pET22b vector and then recombinant 6His-linker domain proteins expressed in *Escherichia coli* and purified in Ni-NTA Sepharose (GE Healthcare). For the *in vitro* kinase experiments, 1 µg 6His-linker domain or the same amount of mutant forms were incubated with 100 µl of HEK293 extracts in the presence of gamma-31[P]-labeled ATP at 37°C. Then the samples were processed for autoradiography or western blot as specified.

### Mutants and cloning

Mutations of serines into alanines were performed on the base of constructs described previously (Tatarskiy et al., 2017). Substitution of serines was performed with primers: 297/301: 5′-ctagacgctgatggtgatgcagatgatggc, 327/331: 5′-gcaatgtagctgaaggggaagcccctcctgac, 323: 5′-gacagctccgctggcaatgta, 335: 5′- cctcctgacgcccaggaggac. For lysine to alanine substitution in the NLS-3 sequence primer 5′-gatggtcgaggtgatgaggcagcaaatgcagg was used. Transfection of purified plasmids into HEK293 was performed with Lipofectamine 2000 (Invitrogen) according to the manufacturer’s protocol.

### Immunoblot and antibodies

For immunoblot analysis, we used polyclonal antibodies against PHF10 described in (Brechalov et al., 2014, 2016) diluted 1:1000 and antibody to α-FLAG (M2 monoclonal antibody, F 1804, Sigma-Aldrich), 1:1000. Immunostaining of FLAG-tagged recombinant PHF10 isoforms in HEK293 cells was performed as described previously (Brechalov et al., 2014), using α-FLAG antibodies (1:100) and secondary anti-mouse Alexa-488 Fluor-conjugated antibodies (A32723; Invitrogen). Stained preparations on glass slides were mounted in mounting medium (Vector Laboratories) and examined under a TCS SP2 confocal microscope. The immunoblots in Fig. 4 were quantified using ImageJ 1.50i (NIH, USA) (Schneider et al., 2012) according to the program's manual.
